# Feto-Maternal Outcomes of Obstructed Labor and Associated Factors among Mothers Who Gave Birth in Public Hospitals of West Shoa Zone, Central Ethiopia: Cross-Sectional Study

**DOI:** 10.4314/ejhs.v31i3.3

**Published:** 2021-05

**Authors:** Alem Getachew, Negash Wakgari, Tolosa Gishille

**Affiliations:** 1 Dire Incini Health Center, West Shoa Zone, Oromia Region, Central Ethiopia; 2 Department of Midwifery, College of Medicine and Health Sciences, Ambo University, Ambo, Ethiopia; 3 Department of Obstetrics and Gynecology, Ambo University Referral Hospitals, Ambo, Ethiopia

**Keywords:** Obstructed labor, maternal outcome, fetal outcome, Central Ethiopia

## Abstract

**Background:**

Obstructed labor the major cause of adverse feto-maternal outcomes. Hence, the study assessed adverse fetomaternal outcomes of obstructed labor and associated factors among women who gave birth in public Hospitals of West Shoa Zone.

**Methods:**

Cross-sectional study design was conducted among 277 respondents. The required data were extracted from the mothers' chart by using a systematic random sampling method. Checklists were used to collect data. Bivariable and multivariable logistic regression was used to identify factors associated with feto-maternal outcomes. The odds ratio with 95%CI and p-value were used to identify the significant variables.

**Results:**

In this study, 145(52.3%) and 157(56.7%) of the respondents had adverse maternal and fetal outcomes respectively. Mothers who were referred from the health center (AOR: 3.96, 95%CI: 1.61–9.8) and who had a trial of labor at the health center and home had a more likelihood of adverse maternal outcomes than those who were referred and had trial of labor at hospital respectively. In addition, mothers who were not followed by partograph and in labor for >24hrs had also a more likelihood of adverse maternal outcomes than their counterparts. Likewise, newborns whom their mothers were not followed by partograph and mothers in labor for >24 hrs had also a more likelihood of adverse fetal outcomes than their counterparts. Hence, health professionals should give special attention to early diagnosis and referral of obstructed labor to higher facilities.

## Introduction

Globally, obstructed labor occurs in an estimated 5% of pregnancies and accounted for 2.8% of all maternal deaths (1). Most of these deaths 99% is being occurred in the developing world (2, 3). In Africa, obstructed labor is responsible for 10.3 to 38.9% of maternal deaths (2, 4, 5).

In Ethiopia, obstructed labor is associated with adverse maternal and fetal outcomes (6–8). Studies conducted in Ethiopia showed that 13–36% of obstructed labor is responsible for all maternal deaths (8, 9). Apart from maternal deaths, obstructed labor had different maternal outcomes such as postpartum hemorrhage (PPH), uterine rupture, puerperal sepsis and recto-vaginal fistula (6–8). Besides of their physical wounds such as fistula, serious social issues of divorce, separation from religious exercises and isolation from their families which might worsen poverty are the major problems of obstructed labor (10–12). In addition, fetal outcomes including stillbirth, birth asphyxia, neonatal sepsis, and neonatal jaundices were also reported (8, 10).

Even though some studies were conducted in Ethiopia on the same issue, but Ethiopia is a multicultural country and there is significant variation in socio-demographic characteristics, delay in receiving quality care at health facilities, availability of equipment, and drugs for emergency obstetric care. Although the particular challenges and complication of obstructed labor varies by settings. Therefore, this study intended to assess the adverse fetomaternal outcomes of obstructed labor and associated factors among mothers who gave birth at West Shoa Zone public Hospitals, Central Ethiopia.

## Materials and Methods

**Study setting, design, and population**: Institution based cross-sectional study was conducted among mothers who gave birth in West Shoa Zone public Hospitals in Oromia National regional state, Central Ethiopia. The study was conducted in seven public Hospitals (Gindabarat General Hospital, Ambo General Hospital, Ambo Referral Hospital, Bako primary Hospital, Inchini primary Hospital, Gedo General Hospital, and Jaldu primary Hospital). All records of mothers presented with obstructed labor and gave birth at West Shoa Zone public hospitals from May 1, 2017, to April 1, 2020, were the study population.

**Study population:** Sample size was determined using a single population proportion formula considering the following assumption: proportion (p) 78% from a previous study (13), 95% confidence interval and 5% margin of error. The final sample size with a 5% non-response rate was 277. The cards of mothers delivered with obstructed labor from May 1, 2017, to April 1, 2020, were identified, separated, and documented from the Health Management Information System, delivery and operation registration books. Based on the number of cases reported in the three years prior to the study, the sample size was proportionally allocated to each hospital and systematic random sampling techniques were employed to select mothers' cards by using the K^th^ value of every 2 intervals.

**Data collection tool and analysis**: Pre-tested and a structured checklist was used to collect the data. The pre-test was conducted among 14 of the sample size to check for consistency and reliability. The results of the pre-test were used to modify the instrument. The checklist was designed to assess socio-demographic characteristics, obstetrics characteristics, health services related factors, provider factors, the maternal and fetal outcome of a mother with obstructed labor. The content of the checklist was adapted from different studies with certain modifications (4, 7, 12–15). The data checked and entered by Epi info and exported to SPSS version 23 for analysis. Bivariable and multivariable logistic regression was used to identify factors associated with adverse fetomaternal outcomes. All explanatory variables having a p-value of less than 0.25 were included in multivariate analysis. AOR with their 95% CI was estimated to identify the presence and strength of associations and statistically significant associations were tested at a p-value of < 0.05.

**The following operational definitions were used.**

**Adverse maternal outcome**: PPH, hysterectomy, uterine rupture, puerperal sepsis, anemia, bladder trauma, perineal tear, cervical tear, cesarean or instrumental vaginal delivery, and maternal deaths. If the mother had one or more of the above conditions (recorded on the chart), she was considered as having adverse maternal outcomes (15–17).

**Adverse fetal outcome**: Birth asphyxia, stillbirth, birth injury, poor Apgar score (Appearance, Pulse, Grimace, Activity and Respiration) and neonatal sepsis. If the newborn had one or more of these conditions (recorded on the chart), considered as having adverse fetal outcome (18). Registered Apgar score of 7–10 is considered as normal Apgar score while 0–6 indicates poor Apgar score (19–20).

**Ethics**: The ethical clearance for this study was obtained from the Institutional review board of Ambo University, College of Medicine and Health Sciences with a reference number of PGC/03/2020. The official supportive letters were provided for each hospital. To collect data permission was obtained from the medical directors of respective hospitals and heads of the maternity ward. The confidentiality of the data was secured throughout the study and information regarding the identification of the mothers was recorded anonymously. To keep the confidentiality their names were not documented; rather a code was given for each card.

## Results

**Social-demographic and Obstetrical characteristics**: Out of the total, 102(36.8%) of the respondents were in the age group of 25–29 years, with the mean age of 26 years. With regard to residence, 207(74.7%) were from a rural area. In addition, 253(91.3%) of them were found in the term gestational age. Further, about 36 (13%) of mothers had ever different obstetrical problems in which hypertension accounts for 16(5.8%) (Table 1).

**Table 1:** Socio-demographic and obstetrical characteristics of mothers with obstructed labor at West Shoa Zone public hospitals, Central Ethiopia, 2020 (n=277)

Variables		Frequency	Percent
Age in years	<20	31	11.2
	20–24	83	30
	25–29	102	36.8
	>30	61	22
Residence	Urban	70	25.3
	Rural	207	74.7
Marital status	Married	238	85.9
	Unmarried	6	2.2
	Unknown	33	11.9
Parity	1	138	49.8
	2–4	95	34.3
	>5	44	15.9
Past obstetric problem	Antepartum hemorrhage	3	1.1
	Premature rupture of membrane	1	0.4
	Preterm labor	3	1.1
	Twin pregnancy	4	1.4
	Hypertension	16	5.8
	Post term pregnancy	3	1.1
	Intrauterine fetal death at admission	6	2.2
Gestational age in weeks	<36	1	0.4
	37–40	253	91.3
	>40	1	0.4
	Unknown gestational age	18	6.5
	Missed data	4	1.4

**Health service-related and provider factors**: About 155(56%) of the mothers did not follow their antenatal care (ANC). Three-forth 205(74%) of them were referred from the health centers. Regarding partograph use 151(54.5%) of mothers were not followed by partographs. About 158(57%) of them were in labor for >24 hrs. Regarding the place of labor trial 159(57.4%) of the respondents were to try labor at the health center (Table 2).

**Table 2:** Health service related factors of mothers with obstructed labor, West Shoa Zone public hospitals, Central Ethiopia, 2020 (n=277)

Variables	Category	Frequency	Percent
**ANC**	Booked	122	44
	Unbooked	155	56
**Partograph plotted**	Yes	126	45.5
	No	151	54.5
**Duration of labor**	≤24 hrs	119	43
	>24 hrs	158	5
**Referral**	Hospital	31	11.2
	Health center	205	74
	Direct coming	41	14.8
**Place of labor trial**	Hospital	62	22.4
	Health center	159	57.4
	Home	56	20.2

**Maternal outcome**: More than half of 145(52.3%) mothers had adverse maternal outcomes (95% CI: 46.6–58.1). Of this sepsis was the commonest unfavorable maternal outcome accounts for 52(18.8%), followed by PPH 28(10.8%), ruptured uterus 17(6.1%), surgical site infection 14(5.1%), postpartum depression 10(3.6%), bladder injuries 3(1.1%) and one case develop a perineal tear.

**Fetal outcome**: Out of the total, 228(82.3%) babies were live birth and 49(17.7%) were stillbirth. Among live birth 157(56.7%) were born with adverse fetal outcomes (95% CI: 50.5–62.5). Among these 53(19.1%) of the newborns had birth asphyxia followed by meconium aspiration syndrome 30(10.8%) and neonatal sepsis 10(3.6%). Apgar score at 5 minutes was found to be normal in 128(46.2%) of the newborns. Admission to the neonatal intensive care unit was 89(32.1%). The weights of the newborns were in the normal range (2500–3999 grams) in 244(88.1%). In addition, early neonatal death accounts for 6(2.2%) and neonatal jaundice found in 5(1.8%) of newborns.

**Factors associated with maternal outcomes of obstructed labor**: In bivariate analysis, source of referral, partograph use, age, duration of labor, parity, place of labor trial, and ANC follow up were found to be significantly associated with adverse maternal outcomes. Source of referral, partograph use, place of labor trial, and duration of labor were found to be significantly associated with adverse maternal outcomes in multivariate logistic regression analysis. The odds of experiencing adverse maternal outcomes for mothers who were referred from health centers were about 4 times compared to mothers who were referred from hospitals (AOR:3.96, 95%CI:1.61–9.8). Regarding the partograph follow up, the odds of experiencing adverse maternal outcomes for mothers who were not followed by partograph was about 2 times compared to those mothers who were followed by partograph (AOR:1.84, 95%CI: 1.09–3.09). Similarly, mothers who had a duration of labor >24hrs had about 2 times more a likelihood of adverse maternal outcome compared to those mothers who had a duration of labor ≤24hrs (AOR: 1.7, 95%CI: 1.03–2.9). In addition, the odds of experiencing adverse maternal outcomes for mothers who were trial of labor at health center was about 2 times compared to those mothers who were trial of labor at hospitals (AOR: 2.4, 95%CI:1.25–4.65). And also, mother who was trial of labor at home had about 3 times more a likelihood of adverse maternal outcome than those mothers who were trial of labor at hospitals (AOR: 2.76, 95%CI:1.22–6.24) (Table 3).

**Table 3:** Bivariate and multivariate logistic regression of factors associated with adverse maternal outcome among mothers with obstructed labor, West Shoa Zone public hospitals, Central Ethiopia, 2020

Variables	No adverse maternal Outcome	Adverse maternal Outcome	COR (95%CI)	AOR (95%CI)
**Referral site**				
Hospital	23(8.3%)	8(2.9%)	1	1
HC	86(31%)	119(43%)	3.98(1.69–93)	3.96(1.61–9.8)*
Direct coming	23(8.3%)	18(6.5%)	2.25(0.82–6.2)	2.6(0.86–7.7)
**ANC follow up**				
Booked	63(22.74%)	59(21.33%	1	1
Unbooked	69(24.9%)	86(31.03%)	1.33(0.83–2.14)	1.3(0.78–7.69)
**Age in years**				
<20	22(7.93%)	9(3.25%)	0.48(0.19–1.18)	0.51(0.2–1.29)
20–24	45(16.25%)	38(13.72%)	1	1
25–29	43(15.5%)	59(21.3%)	1.6(0.91–2.9)	1.32(0.7–2.47)
>30	22(8%)	39(14.05%)	2.1(1.1–4.14	1.7(0.83–3.5)
**Place of labor trial**				
Hospital	40(14.45%)	22(7.94%)	1	1
Health center	69(24.91%)	90(32.5%)	2.37(1.29–4.4)	2.4(1.25–4.65)*
Home	23(8.3%)	33(11.9%)	2.61(1.24–5.49)	2.76(1.22–6.24)*
**Partograph follow up**				
Yes	70(25.3%)	56(20.22%)	1	1
**No**	62(22.38)	89(32.1%)	1.79(1.1–2.9)	1.84(1.09–3.09)*
**Duration of labor**				
<24hrs	72(26%)	59(21.3%)	1	1
>24hrs	60(21.7%)	86(31%)	1.75(1.09–2.82)	1.7(1.03–2.9)*

* Significant variables in multivariate logistic regression analysis

**Factors associated with fetal outcome**: Partograph follow-up, age, duration of labor, parity, residence, and place of labor trial were significantly associated with adverse fetal outcome in bivariate logistic regression analysis. From this partograph follow up and duration of labor were found to be significantly associated with adverse fetal outcome in multivariate logistic regression analysis. The odds of experiencing unfavorable fetal outcomes for mothers who had a duration of labor >24hrs was about 2 times compared to those mothers who had duration of labor ≤24hrs (AOR: 1.72, 95%CI: 1.05–2.80). Similarly, the odds of experiencing adverse fetal outcomes for mothers who were not followed by partograph was about 2 times compared to those mothers who were followed by partograph (AOR:1.79,95%CI: 1.01–2.93) (Table 4).

**Table 4:** Result of bivariate and multivariate logistic regression on factors associated with adverse fetal outcome among mothers with obstructed labor at West Shoa Zone public hospitals, Oromia, Ethiopia, 2020

Variables	No adverse fetal outcome	Adverse fetal outcome	COR (95%CI)	AOR((95%CI)
**Residence**				
Urban	36(13%)	34(12.3%)	1	1
Rural	84(30.3%)	123(44.4%)	1.55(0.89–2.7)	1.59(0.92–2.79)
**Parity**				
Para 1	62(22.4%)	76(27.43%)	1.01(0.60–1.7)	1.01(0.58–1.75
Para 2–4	43(15.5%)	52(18.77%)	1	1
Para >5	15(5.4%)	29(10.5%)	1.59(0.76–3.36)	1.5(0.68–3.22)
**Place of labor trial**				
Hospital	30(10.8%)	32(11.6%)	1	1
Health center	63(22.74%)	96(34.66%)	1.43(0.79–2.58)	1.52(0.82–2.8)
Home	27(9.74%)	29(10.46%)	1.01(0.49–2.08)	1.02(0.48–2.18)
**Duration of labor**				
<24hrs	66(23.83%)	65(23.47%)	1	1
>24hrs	54(19.5%)	92(33.2%)	1.73(1.07–2.79)	1.72(1.05–2.80)*
**Partograph follow up**				
Yes	64(23%)	62(22.38%)	1	1
No	56(20.22%)	95(34.3%)	1.75 (1.08–2.8)	1.79(1.01–2.93)*
**Age in years**				
<20	11(4%)	20(7.22%)	2.15(0.92–5.05)	2.14(0.896–5.1)
20–24	45(16.24%)	38(13.72%)	1	1
25–29	44(15.9%)	58(20.9%)	1.56(0.87–2.79)	1.35(0.74–2.47)
>30	20(7.22%)	41(14.8%)	2.43(1.2–4.83)	2.03(1.01–4.13)

* Significant variables in multivariate logistic regression analysis

## Discussion

In this study, more than half 145(52.3%) of mothers had adverse maternal outcome (95% CI: 41.9–53.1). This finding is in line with studies conducted in Kashmir India (48.7%) (22), Madhya Pradesh India (50.5%) (23), and Pakistan (52.95%)(24). However, the present result is lower than studies done in Jimma (68.2%) (25), Suhul hospital (88.6%) (7), Bangladesh (76.19%) (26), and higher than the studies conducted in Nigeria (37.56%) (4), Sudan (14.3%) (27), and West Bengal, India (33.9%) (28). This might be due to differences in socio-demographic characteristics, infrastructures, health care services and time of the study (29–30).

Likewise, 56.7% of the newborns delivered from mothers with obstructed labor had adverse fetal outcomes (95% CI: 50.5–62.5). This figure is consistent with the studies conducted in Nigeria (61.4%) (4) and Bangladesh (51.31%) (26). However, it is lower than the studies conducted in India (76.5%) (22), Mettu (78.5%) (10), and higher than the study conducted in Wollega (14). The possible explanation for this difference might be differences in sample size and study setting. For instance, the sample size was 402 in the study of India and 143 in Wollega's study. Moreover, the difference in the management of obstructed labor, source of referral, distance from the health facility and access to transportation could also reason for the gap.

Mothers who were referred from the health centers had a more likelihood of adverse maternal outcomes. This is consistent with the study conducted in Mettu (10). This is because majority of mothers were rural residents, which may predispose to long distance travel to health facility and later arrival after complication happen. Moreover, mothers who had trial of labor at the health center and home had a more likelihood of unfavorable maternal outcome compared to those who had at hospitals. This result is supported by study conducted in India (23). This might be due to those mothers who had trial of labor at hospitals were near to access quality comprehensive emergency obstetric and newborn care service than those mothers who had trial of labor at health center and home (29).

Furthermore, mothers who were not followed by partograph had more a likelihood of adverse maternal outcome than the counterpart. This finding is in line with the study done in Wollega (14). This is due to the fact that partograph is often used to track labour progress and diagnose complications early (31). Similarly, mothers who was in labor for >24hrs had a more likelihood of unfavorable maternal outcome. This is consistent with the studies done in Mizan-Aman Hospital and Mettu (10, 32).

In this study, mothers who were not followed by partograph had a more likelihood of adverse fetal outcome. This is similar to the study done in Wollega (14). This is well explained by purpose of partograph use to monitor fetal conditions (31). Similarly, a labor duration of >24hrs had also a more likelihood of unfavorable fetal outcome. This is in line with the study conducted in Bangladesh (33) and Mizan-Aman Hospital (32). This is because as duration of labor increase without progress of labor the baby not get enough oxygen, which may result in death and unfavorable fetal outcome (34). In this study, unfavorable fetomaternal outcome is found high. Source of referral, partograph use, place of labor trial and duration of labor had significantly associated with unfavorable maternal outcome. In addition, duration of labor and mothers not followed by partograph was also significantly associated with unfavorable fetal outcome. Health professionals should give special attention on early diagnosis and referral of obstructed labor to higher facility. Due to Covid-19 situation one hospital is excluded from the study.
